# An Analysis of the Neutralizing Antibodies against the Main SARS-CoV-2 Variants in Healthcare Workers (HCWs) Vaccinated against or Infected by SARS-CoV-2

**DOI:** 10.3390/vaccines11111702

**Published:** 2023-11-08

**Authors:** Palmira Immordino, Vincenzo Pisciotta, Emanuele Amodio, Celestino Bonura, Floriana Bonura, Federica Cacioppo, Giuseppe Calamusa, Giuseppina Capra, Alessandra Casuccio, Simona De Grazia, Dario Genovese, Davide Graci, Guido Lacca, Giuseppa Luisa Sanfilippo, Maria Gabriella Verso, Giovanni Maurizio Giammanco, Donatella Ferraro

**Affiliations:** Dipartimento di Scienze per la Promozione della Salute e Materno Infantile “G. D’Alessandro”, PROMISE, Università di Palermo, 90127 Palermo, Italy; vincenzo.pisciotta@unipa.it (V.P.); emanuele.amodio@unipa.it (E.A.); celestino.bonura@unipa.it (C.B.); floriana.bonura@unipa.it (F.B.); federica.cacioppo@unipa.it (F.C.); giuseppe.calamusa@unipa.it (G.C.); giuseppina.capra@unipa.it (G.C.); alessandra.casuccio@unipa.it (A.C.); simona.degrazia@unipa.it (S.D.G.); dario.genovese@unipa.it (D.G.); davide.graci01@unipa.it (D.G.); guido.lacca@unipa.it (G.L.); giuseppaluisa.sanfilippo@unipa.it (G.L.S.); mariagabriella.verso@unipa.it (M.G.V.); giovanni.giammanco@unipa.it (G.M.G.); donatella.ferraro@unipa.it (D.F.)

**Keywords:** SARS-CoV-2, healthcare workers, vaccine, COVID-19, neutralizing antibodies, variants of concern

## Abstract

Although the anti-COVID-19 vaccination has proved to be an effective preventive tool, “breakthrough infections” have been documented in patients with complete primary vaccination courses. Most of the SARS-CoV-2 neutralizing antibodies produced after SARS-CoV-2 infection target the spike protein receptor-binding domain which has an important role in facilitating viral entry and the infection of the host cells. SARS-CoV-2 has demonstrated the ability to evolve by accumulating mutations in the spike protein to escape the humoral response of a host. The aim of this study was to compare the titers of neutralizing antibodies (NtAbs) against the variants of SARS-CoV-2 by analyzing the sera of recovered and vaccinated healthcare workers (HCWs). A total of 293 HCWs were enrolled and divided into three cohorts as follows: 91 who had recovered from SARS-CoV-2 infection (nVP); 102 that were vaccinated and became positive after the primary cycle (VP); and 100 that were vaccinated with complete primary cycles and concluded the follow-up period without becoming positive (VN). Higher neutralization titers were observed in the vaccinated subjects’ arms compared to the nVP subjects’ arms. Differences in neutralization titers between arms for single variants were statistically highly significant (*p* < 0.001), except for the differences between titers against the Alpha variant in the nVP and in VP groups, which were also statistically significant (*p* < 0.05). Within the nVP group, the number of subjects with an absence of neutralizing antibodies was high. The presence of higher titers in patients with a complete primary cycle compared to patients who had recovered from infection suggested the better efficacy of artificial immunization compared to natural immunization, and this further encourages the promotion of vaccination even in subjects with previous infections.

## 1. Introduction

At the end of 2019, the first cases of severe acute respiratory syndrome coronavirus-2 (SARS-CoV-2) infection were diagnosed. Since the first few months of 2020 and over the course of 2021, SARS-CoV-2, which belongs to the Betacoronavirus family, has demonstrated its ability to provoke a debilitating condition known as COVID-19 (corona virus disease 2019). This novel disease was primarily characterized by a constellation of symptoms reminiscent of an influenza-like syndrome, comprising fever, a persistent dry cough, shortness of breath, and the distressing symptom of dyspnea. Moreover, this viral illness is often accompanied by a panoply of additional symptoms, which may encompass manifestations such as headaches, dizziness, episodes of vomiting, and bouts of diarrhea [1,2]. In some cases, SARS-CoV-2 infections may present various forms of mildly symptomatic affections or even acute respiratory distress syndromes (ARDS), accompanied by a severe systemic inflammation, leading eventually to multiple organ failure (MOF) [1].

Since the very beginning of the pandemic, it was evident that vulnerability to the infection and the degree of severity in clinical manifestations were intricately linked to the ages of patients. In general, older individuals with preexisting comorbidities were more predisposed to experiencing severe symptomatic infections which could progress to the extent of necessitating hospitalization and, in the worst cases, even resulting in mortality. Conversely, younger subjects have tended to manifest milder forms of the illness or, in some instances, remain entirely asymptomatic despite being infected [3,4].

As for other Betacoronaviruses, human-to-human transmission happens through respiratory droplets. Their median incubation time is four to six days before the onset of symptoms, and during this period, high viral loads have been identified in hosts or at the symptoms’ onset and during the first week of the disease, determining a high transmission potential even with mildly symptomatic infections [1,3]. These have led to the rapid spread of SARS-CoV-2 worldwide, causing more than 100,000 confirmed cases and over 4000 deaths by early 2020 [5]. Therefore, on 11 March 2020, COVID-19 was declared a pandemic by the WHO Director-General [5], launching a global effort to develop effective vaccines [6].

In a remarkably short timeframe, starting as early as the year 2020, we witnessed the rapid development of multiple vaccines with the intent of not only pursuing but also successfully attaining an effective tool for preventing SARS-CoV-2 infections [1]. Among these pioneering efforts, the BNT162b2 mRNA COVID-19 vaccine, known as Comirnaty^®^, stood out as an mRNA-based vaccine designed to encode the entire SARS-CoV-2 spike glycoprotein (S). It achieved the distinction of being the very first SARS-CoV-2 vaccine to gain approval by both the Food and Drug Administration (FDA) and the European Medicines Agency (EMA) in December 2020. At the end of the same month, the vaccination campaign against SARS-CoV-2 was launched, ushering in a new phase in our global response to the pandemic. This initiative swiftly demonstrated its efficacy, having an immediate and positive impact on the prevalence of severe illness associated with COVID-19, providing hope and protection to countless individuals. Several studies have demonstrated the immunogenicity of the Comirnaty ^®^ vaccine, showing a sustained antibody response after vaccination. Originally, the primary vaccination schedule included the injection of two doses of BNT162b2 21 days apart. A level of 95% protection against COVID-19 has been claimed following the administration of two doses of BNT162b2 [7]. Despite these results, several people have been infected by SARS-CoV-2 after a second dose of BNT162b2, as has been demonstrated through an RT-PCR molecular test on a nasopharyngeal swab (NPS) sample.

Most of the SARS-CoV-2 neutralizing antibodies (NtAbs) produced after SARS-CoV-2 infection target the spike (S) protein receptor-binding domain (RBD) [8,9,10]. The RBD is a subdomain of the SARS-CoV-2 S-protein, which is exposed on the virus membrane and has an important role in facilitating viral entry and the infection of the host cells [9,10]. SARS-CoV-2 has demonstrated a remarkable capacity to evolve, primarily driven by the accumulation of mutations within its spike protein. This mutation process has helped the virus in its endeavor to escape the humoral response of a host’s immune system, thereby presenting a formidable challenge. As a consequence, we have observed the rapid emergence of SARS-CoV-2 variants of concern (VOC), including but not limited to the Alpha variant (B.1.1.7), the Gamma variant (P.1), the Delta variant (B.1.617.2), and the most recent addition, the Omicron variant (B.1.1.529) [11]. These variants have proven to be highly disruptive, causing substantial and widespread outbreaks even within populations that had previously experienced infections or had been vaccinated. These dynamics underscore the need for continued vigilance and adaptability in our response to the evolving SARS-CoV-2 virus.

Since the early months of the pandemic’s outbreak, it has been starkly evident that healthcare workers face a considerable and noteworthy risk of contracting SARS-CoV-2 infections, along with the associated burden of morbidity and mortality [12,13]. This risk has consistently proven to be notably higher when compared to the general population, especially for those dedicated professionals on the frontline of healthcare services, whether they were provided with adequate personal protective equipment or not [14]. This has led to prioritizing healthcare workers’ vaccinations at the start of the anti-COVID immunization campaigns worldwide, alongside elderly patients [15]. Healthcare workers’ exposures were shown to be related to a considerable, although declining, risk of SARS-CoV-2 infection throughout the year 2020 and during the early phases of the vaccination campaign in 2021 [16].

Keeping these premises in mind, the aim of the present study was to identify factors that might have a role in SARS-CoV-2 infections in vaccinated healthcare workers (HCWs) by analyzing sera from different subject groups.

With the objective of understanding why some vaccinated HCWs were infected, we compared vaccinated subjects’—and not vaccinated infected subjects’—sera and measured the antibodies’ neutralization capacities against wild-type SARS-CoV-2 and other variants using an in vitro live virus neutralization assay.

## 2. Materials and Methods

### 2.1. Population

We retrospectively analyzed the differences in the neutralizing antibodies’ responses in HCWs of both sexes that were aged between 18 and 70 and worked at the Policlinico University Hospital “P. Giaccone” of Palermo. These HCWs voluntarily requested to be tested for SARS-CoV-2 serology at the OU of the Microbiology and Virology department of the same hospital, and their sera were collected from October 2020 to October 2021 at the Microbiology and Virology Laboratory of the University Hospital.

**Figure 1:**
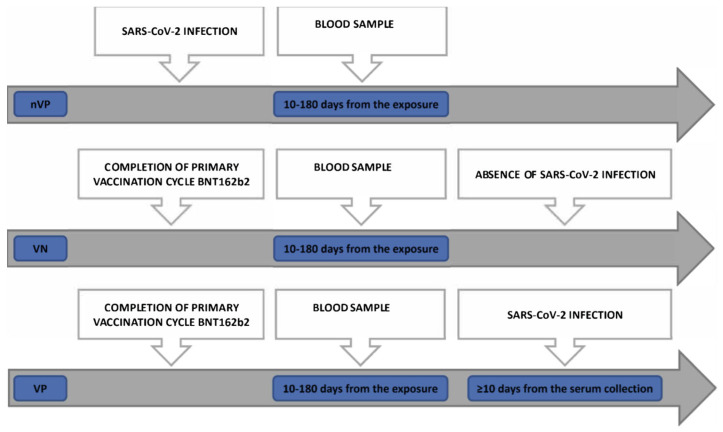
Inclusion criteria for the three study cohorts and their respective timelines.

Our study population was divided into 3 cohorts of HCWs (Figure 1) as follows:

1. A non-vaccinated positive (nVP) cohort: unvaccinated subjects who were diagnosed as being positive for SARS-CoV-2 infection by molecular tests on nasopharyngeal swab (NPS) samples between August 2020 and March 2021 and whose sera were collected 10 to 180 days after the positive tests for COVID-19
2. A vaccinated positive (VP) cohort: subjects who received 2 doses of BNT162b2 and were successively diagnosed as being positive for SARS-CoV-2 using RT-PCR molecular tests with NPS samples and whose sera were collected 10 to 180 days from administration of the second dose of vaccine, encompassing the humoral response peak and the decline of the humoral response [17,18], and at least 10 days before any diagnosis of infection (“positivization”) with the aim of avoiding evaluating the neutralization mediated by the antibodies elicited during an early asymptomatic phase of SARS-CoV-2 infection
3. A vaccinated non-positive (VN) cohort: subjects who received 2 doses of BNT162b2, without evidence of post-vaccination SARS-CoV-2 infections, with sera collected 10 to 180 days after the second vaccine dose administration

We identified the period for sera collection as 10 to 180 days after exposure in the inclusion criteria with the aim of not losing the opportunity to include subjects who spontaneously underwent blood withdrawal after several months from the second dose administration, while paying attention to the decline in natural antibodies that has largely been described in the literature. In fact, it is known that after six months, antibody titers decrease, and so analyzing sera collected after this period could have shown extremely low titers that could have been mistaken for a lack of post exposure immunization, being instead due the above-mentioned phenomenon [17,18,19].

Figure 1 describes the three different cohorts and summarizes the main phases, with the relative time period in between, in days, for each group. It shows the exposure moment (whether infection or primary cycle vaccination), the blood collection moment, and the infection moment after vaccination, if present.

Subjects included in the three different cohorts were matched by age (±5 years), sex, and days (±15) to blood collection from the administration of a vaccine for the VN and VP groups or days (±15) from blood collection to a positive COVID-19 test for the nVP group. The date of COVID-19 positivity was retrieved from the national COVID-19 cases database, which had been updated until February 2022.

### 2.2. Virus Neutralization Assay

We used a previously described in vitro live virus neutralization assay to quantify the neutralizing antibodies (NtAbs) [20]. The Vero E6 cell line, cultured and maintained at 37 °C in MEM containing 10% FBS and antibiotics (100 U/mL of penicillin and 100 μg/mL of streptomycin) (Gibco, Life Technologies, Waltham, MA, USA), was used to isolate the wild-type SARS-CoV-2 and its VOCs (the Alpha, Delta, Gamma, and Omicron variants), which were obtained from clinical samples. The isolation and live virus micro-neutralization assays were performed in a BioSafety Level (BSL) 3 laboratory. The genetic characteristics of the SARS-CoV-2 strains were investigated by sequence analysis of the spike proteins before and after isolation in cell cultures. The testing procedures for the clinical specimens were carried out through strict observance of the WHO interim guidance [21]. The titers of the NtAbs were determined using cells that were separately infected with the wild-type virus and the above-mentioned VOCs. The degree of cytopathic effect (CPE) was evaluated after 3 days of incubation at 37 °C in 5% CO_2_. The NtAbs titers were defined as the reciprocal values of the sample dilutions (from 1:10 to 1:1280) showing 50% protection from the virus-induced cytopathic effect (ID50). Titers below 10 were reported as “negative”.

### 2.3. Statistical Analysis

The categorical variables were summarized by frequencies and relative frequencies (%), whereas the continuous variables were shown as means (standard deviations (SDs)) if normally distributed and medians (interquartile ranges (IQRs)) for not normally distributed variables. Moreover, the neutralizing antibody titers were summarized by geometric means and geometric standard deviations (GSDs), in accordance with the international scientific literature [22]. To test for the normal distribution of the continuous data, the Shapiro–Wilk test for normality was applied. To compare the demographic data among the groups, Pearson’s chi-squared test was used. Inter-group comparisons were assessed by using the Mann–Whitney U test.

All statistical analyses were conducted using R for Statistical Computing (R version 4.2.1, Vienna, Austria) within the Rstudio interface (Rstudio, PBC, Boston, MA, USA), and a *p*-value of < 0.05 was considered statistically significant [23,24].

## 3. Results

The study population comprised 293 HCWs, of which 91 were in the nVP cohort, 102 were in the VP cohort, and 100 were in the VN cohort. Their demographic characteristics are represented in Table 1.

During the observation period, some subjects enrolled in the VN cohort became SARS-CoV-2-positive, and therefore, they were moved to the VP cohort, resulting in a total number of 102 HCWs in this cohort. Their presence did not lead to any statistically significant differences for sex and age between the three cohorts. We then replaced the missing subjects in the VN cohort with other HCWs with the same characteristics.

The number of female subjects in the nVP cohort was 56 (61.5%), and there were 59 (57.8%) women in the VP cohort and 62 (62%) in the VN cohort. The differences in sex distribution among the groups were not statistically significant. Also, the median age (36 in the nVP group, 32 in the VP group, and 33.5 in the VN group) and the median number of days to blood collection from vaccination or exposure to the infection (61 for the nVP group, 51 for the VP group, and 57.5 for the VN group) showed no statistically significant differences between the cohorts.

Table 2 shows the general serological characteristics of the three population groups. In the nVP cohort, the geometric mean NtAbs titers ranged from 5.92 against the Omicron variant to 24.11 against Alpha; in the VP cohort, it ranged from 10.50 against Omicron to 48.45 against Gamma; and in the VN cohort, it ranged from 7.45 against Omicron to 60.90 against Alpha. Considerable proportions of the sera analyzed did not have detectable NtAbs against the Omicron variant (76.92% in the nVP group, 42.16% in the VP group, and 56% in the VN group).

**Figure 2:**
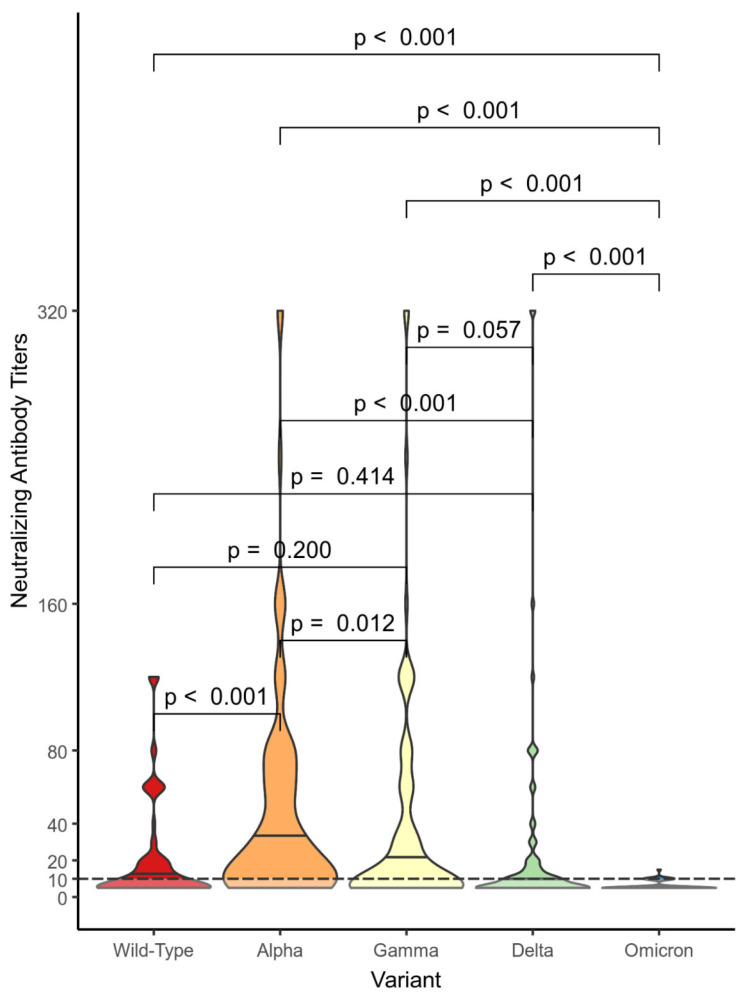
Comparison between the neutralizing antibody titer distributions against the different variants within the unvaccinated positive subjects (nVP) cohort. We used the Mann–Whitney U test with the associated *p*-values.

**Figure 3:**
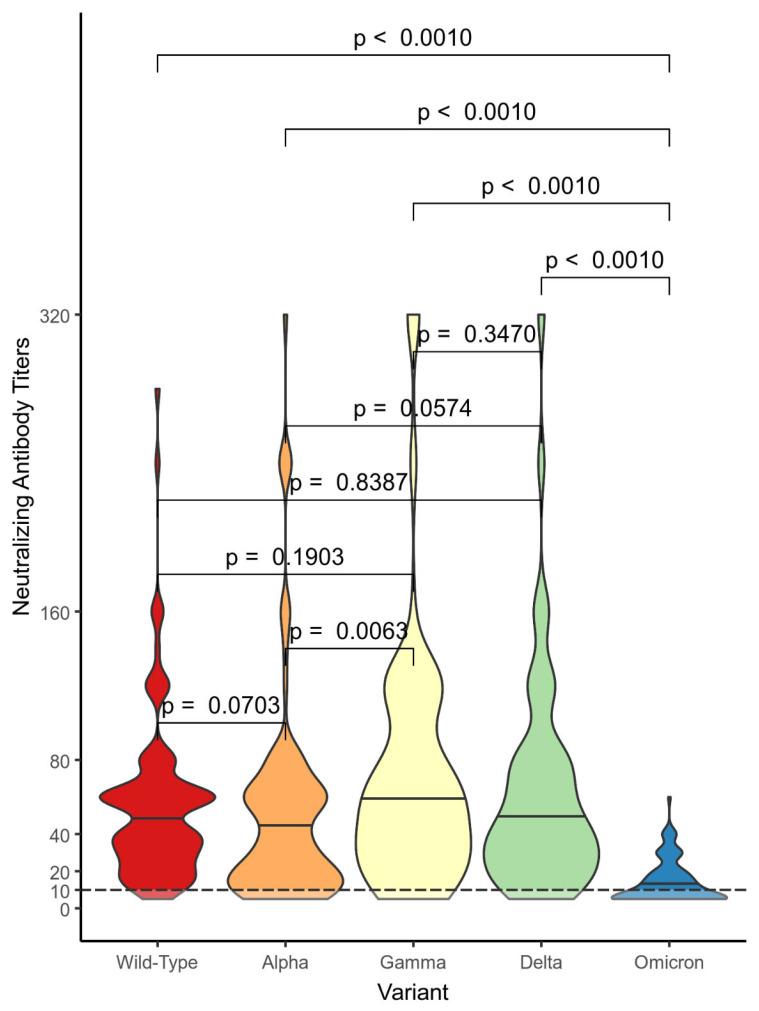
Comparison between the neutralizing antibody titer distributions against the different variants in the vaccinated positive subjects (VP) cohort. We used the Mann–Whitney U test with the associated *p*-values.

**Figure 4:**
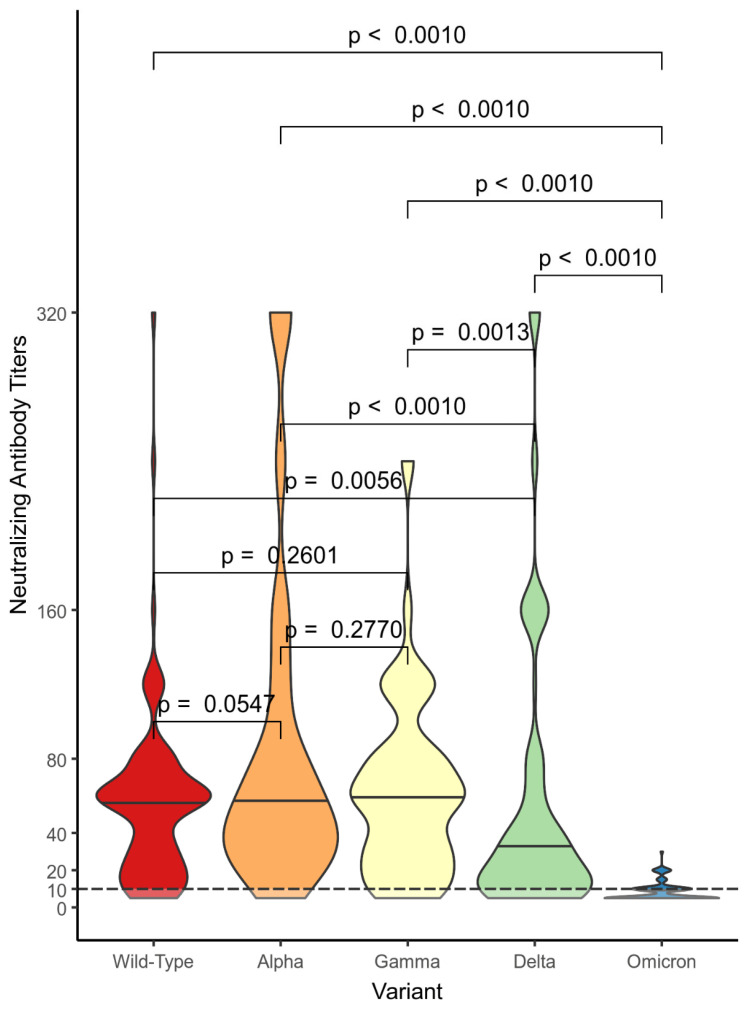
Comparison between the neutralizing antibody titer distributions against the different variants in the vaccinated non-positive (VN) cohort. We used the Mann–Whitney U test with the associated *p*-values.

Figure 2, Figure 3 and Figure 4 show violin plot graphs representing the distributions of the NtAbs titers against the wild-type SARS-CoV-2 and VOCs for each group (nVP—Figure 2, VP—Figure 3, and VN—Figure 4). The subjects in the nVP cohort showed a wide range of NtAbs titers (0 to 320) against the Alpha, Gamma, and Delta VOCs and a shorter range against the wild-type variant, while the NtAbs against Omicron were almost undetectable (0 to 15). In the nVP group, the comparison between the NtAbs titer distributions showed that almost all differences were statistically significant, and we found no statistically significant differences when only comparing the distributions of the NtAbs titers against the wild-type variant versus those against Gamma (*p* = 0.20) and, similarly, for the distributions of the NtAbs against the wild-type variant versus those against Delta (*p* = 0.41) (Figure 2).

Subjects in the VP cohort showed the following distributions ranges for the NtAbs against the VOCs: from 0 to 280 against the wild-type variant; from 0 to 320 against Alpha, Gamma, and Delta; and from 0 to 60 against Omicron. In the VP group, the differences were statistically significant in comparison to the NtAbs titer distributions against Alpha and against Gamma (*p* = 0.006) and in comparison to the NtAbs titer distributions against Omicron and against each of the other variants. Other comparisons for the VP group did not show differences that were statistically significant (Figure 3).

Subjects in the VN group had the following distribution ranges for the NtAbs titers: from 0 to 320 against the wild-type variant, Alpha, and Delta; from 0 to 240 against Gamma; and from 0 to 40 against Omicron. In the VN group, almost all the differences between the NtAbs titer distributions were statistically significant. Only comparisons between the NtAbs titer distributions against the wild-type variant and against Alpha (*p* = 0.0547), against the wild-type and against Gamma (*p* = 0.26), and against Alpha and against Gamma (*p* = 0.277) were not statistically significant (Figure 4).

The differences in the NtAbs titer distributions against Omicron and against the other VOCs were statistically significant in all three groups.

**Figure 5:**
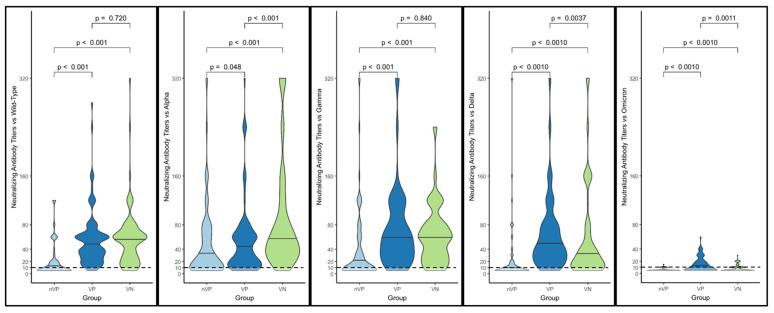
Comparisons between distributions of NtAbs titers in each group against each different variant. Almost all differences in the NtAbs distributions for each variant are statistically significant.

In Figure 5, the violin plot graph describes the comparisons between the distributions of the NtAbs titers in each group against each different variant. Almost all differences in the NtAbs distributions for each variant were statistically significant.

The only differences that were not statistically significant were those between the NtAbs titer distributions against the wild-type variant in the VP and VN groups (*p* = 0.72) and those between the NtAbs titer distributions against Gamma in the VP and VN groups (*p* = 0.84).

## 4. Discussion

Our study aimed to evaluate the possible determinants of infection in subjects already immunized against SARS-CoV-2 either by a previous infection or by vaccination. HCWs were chosen as the target population of our study because they have been considered at high risk for SARS-CoV-2 infection, morbidity, and mortality since the beginning of the pandemic spread [12,14]. For the same reason, they were identified as a priority target group in the earliest phases of the vaccination campaigns [15]. In 2021, new variants of SARS-CoV-2 emerged and spread rapidly, with different infectivity and transmissibility patterns, leading first to a replacement of the wild-type SARS-CoV-2 strain and then, in turn, becoming the predominant strain responsible for the subsequent “pandemic waves” [20]. Therefore, with the aim of speculating on vaccine efficacy against future variants, we assessed the neutralizing efficacy of the immune response against the major SARS-CoV-2 VOCs compared to the wild-type strain, whose spike protein mRNA was included in the vaccine [25,26,27,28].

The presence of specific NtAbs has been predictive of adequate protection from SARS-CoV-2 infection by both the wild-type variant and the VOCs, although a decline in neutralizing titers against VOCs has been described elsewhere [28,29]. Many studies have described the capacity of the wild-type strain of SARS-CoV-2 and other VOCs to escape the protective host immunity induced by both natural infection and vaccination [29,30,31,32,33,34,35,36]. Overall, the analysis of the HCWs’ sera showed a generally acceptable neutralizing potency when compared to results available in the literature [29,37].

In our study, subjects in the nVP cohort showed higher titers against the Alpha variant than those against all other strains. The prevalence of Alpha-specific NtAbs was not surprising as that variant predominantly circulated during the time period in which the nVP population was infected [38], but the lack of protection from subsequent VOCs somewhat confirmed the escape ability gained by the newer variants. On the other hand, vaccinated individuals developed comparable mean neutralizing responses to all the VOCs.

As expected, a higher proportion of subjects who did not receive the vaccine did not produce measurable levels of NtAbs. A number of studies in the literature have described that the wild-type SARS-CoV-2 strain and other variant strains may escape a host’s protective immunity induced by both natural infection and by vaccination [32,34,35,36].

The remarkable difference between the anti-Alpha NtAbs titers and the anti-Omicron NtAbs titers (which showed significantly lower or absent antibody production) in the nVP cohort somewhat confirmed the escaping ability of this variant. The prevalence of Alpha-specific NtAbs might have been attributable to the circulation of that variant during part of the time range in which the nVP population had been infected. An increasing incidence of infections by the Alpha VOC, although never dominating, was characterized by a wave of COVID cases during the last weeks of 2020 and the first months of 2021 in Sicily [38], and this might explain our results. Such a hypothesis can only be presumed but not confirmed due to the unavailability of genotyping on the biological samples.

However, in our study, the neutralizing response against different viral strains was encouraging, in general, and all the groups showed wide ranges of NtAbs titers against the wild-type variant and the VOCs, with the exception of the Omicron strain. Antibodies against the latter were significantly lower in all groups, and a remarkable number of subjects did not have detectable levels of NtAbs against the Omicron strain. This result aligned with previous studies that described considerably reduced humoral responses against the Omicron strain and almost no vaccine effectiveness against symptomatic disease after two doses of BNT162b2 [39]. Omicron sub-variants have generally higher immune escape ability than other SARS-CoV-2 VOCs [35,40], even though the earlier Omicron sub-variants (BA.1 and BA.2) appeared to have stronger binding affinities than other VOCs [40]. The Omicron variant is characterized by great antigenic variability due to the selection of 37 mutations in the spike protein, the main immunogenic protein of the virus, and it is capable of inducing the production of neutralizing antibodies [41,42]. However, significant protection against Omicron has been shown at least within a short time after a booster dose, but the initial elevation in the NtAbs titers was followed by a remarkable decline over time [39,43,44]. These results raised concerns about the potential protective efficacy of antibodies against Omicron over time [45,46], justifying the need for the periodic administration of booster doses and the production of vaccines based on newly emerging variants. This was further confirmed by a recent literature review that analyzed several studies and implied how booster dose administration increased NtAbs production and conferred stronger protection against severe COVID-19 [47].

The analysis of the HCWs’ sera collected in this study showed a generally acceptable neutralizing potency, confirming the results of several studies that focused on the immune response to SARS-CoV-2 infection and/or COVID-19 vaccination [20,31]. However, a significantly lower neutralizing response following natural infection was recorded in the unvaccinated group (nVP) compared to the vaccinated HCWs, once again confirming the importance of promoting these professionals’ immunization, keeping in mind that their role exposes them to a higher risk of infection than the general population and to becoming a potential vehicle for virus transmission to subjects with comorbidities and weaker immune systems.

It is worth noting that to underline that our study, due to its focus on a sample of healthcare workers (HCWs) who were inherently more exposed to a wide spectrum of infectious agents in their occupational settings, may exhibit limitations in terms of the generalization of its findings to the broader population. On the other hand, it is important to consider that the number of unvaccinated healthcare workers is rapidly diminishing. This reduction is attributable to the enforcement of mandatory SARS-CoV-2 vaccinations for such professionals, as instituted by an emergency decree in Italy on 1 April 2021. Consequently, the future recruitment of additional participants into the non-vaccinated HCW cohort is poised to become progressively more challenging as time passes.

It has been estimated that lower titers are required for protection from severe infection than the ones required to protect from any symptomatic infection and mild/moderate symptomatic infection, and so it is possible to assert that despite the decrease in immunity after months from an exposure and the consequent lower protection from infection, vaccinated people still have efficient protection from SARS-CoV-2′s severe forms [29]. Unfortunately, it was not possible to collect information on the clinical statuses of the infected participants in both the nVP and VP groups, limiting our ability to correlate humoral immunity with the development of symptomatic infection. Lastly, another limitation of our study was that it did not examine other immune responses (e.g., innate or cellular immunity) which are commonly involved in contrasting viral infections and are elicited by COVID vaccination [18], preventing us from assessing any potential correlation between the different types of immune responses.

Despite this, it is critical to underline that these data underscore the need for promoting COVID-19 vaccination on a routine basis. This is due to the emergence of novel VOCs, as well as evidence indicating that antibody protection conferred by vaccinations based on SARS-CoV-2 variations genetically distant from the circulating version is insufficient. Recent advancements have resulted in the approval of a vaccine formulation based on mRNAs encoding the SARS-CoV-2 VOC XBB.1.5′s spike protein [48], and this Omicron sub-lineage has been shown to be resistant to the immune response induced by one of the latest vaccines based on both the wild-type variant and Omicron BA.4/BA.5 [48,49]. Based on these findings and a plethora of additional information, it is the role of professionals working in public health to educate the general population about the benefits of having SARS-CoV-2 vaccinations in the following months and to maintain an active surveillance system to detect the emergence of new VOCs, evaluate the risks for communities, and be prepared to limit the undesired spread of the virus, all of which are aimed at preventing increases in the numbers of patients suffering from respiratory diseases caused by SARS-CoV-2 infections.

## 5. Conclusions

The findings and outcomes derived from the current study provide additional and valuable evidence to firmly establish the effectiveness of antibodies in safeguarding individuals from the prevalent VOCs of the SARS-CoV-2 virus.

This protection is confirmed to be valid for a diverse range of individuals, encompassing both those who have not yet received any COVID-19 vaccinations (unvaccinated subjects) and those who have been vaccinated against the virus (vaccinated subjects). However, the humoral responses of people who have been infected and those of unvaccinated people appeared to be less adequate for contrasting the different variants of SARS-CoV-2. Therefore, continued future research might be accompanied by an investigation of the clinical features of SARS-CoV-2 infection in immunized subjects. The procedures illustrated in this paper could be applied to evaluate the several vaccine platforms available and those that may be developed in the future, as well as their immunizing potential. Our findings confirmed the goodness of the choice to promote the administration of the first booster dose to both previously infected and vaccinated people. Moreover, it is crucial to take into consideration the fact that the level of immunity against the Omicron variants, which is challenged by both prior infection and vaccination, requires the ongoing and vigilant surveillance of the circulation of VOCs. This ongoing monitoring is essential not only for the identification of newly emerging SARS-CoV-2 variants but also for the development of robust and effective new vaccines that are designed specifically to combat and mitigate the transmission and proliferation of these emerging variants in the population. These results are extremely important and decisive for encouraging the periodic administration of further updated vaccine doses, with proper public health measures.

It could be interesting in the future to make the same comparisons considering the administration of updated vaccines and considering the new variants that have emerged in recent months, as well as those that will emerge in the future.

## Figures and Tables

**Table 1:** The sociodemographic characteristics of the three study cohorts (nVP (the not vaccinated positive cohort), VP (the vaccinated positive cohort), and VN (the vaccinated non-positive cohort)).

	nVP	VP	VN	*p*-Value
No. of Subjects	91	102	100	
Sex, N (%)				
- Female	56 (61.5%)	59 (57.8%)	62 (62%)	0.804
- Male	35 (38.5%)	43 (42.2%)	38 (38%)	
Age in years, median (IQR)	36 (29–51.5)	32 (27–51)	33.5 (28–51)	0.485
Days to serum collection from exposure, median (IQR)	61 (31.5–102.5)	51 (18.3–995)	57.5 (18.8–104)	0.140

**Table 2:** The serological characteristics of the three study cohorts (nVP (the not vaccinated positive cohort), VP (the vaccinated positive cohort), and VN (the vaccinated non-positive cohort)).

	nVP	VP	VN
Neutralizing antibody titers against the variants (reciprocal value of the sample dilution), geometric mean (±GSD)			
- Wild-type	11.68 (±2.72)	43.92 (±2.11)	41.10 (±2.42)
- Alpha	24.11 (±3.40)	33.25 (±2.71)	60.90 (±2.67)
- Delta	10.55 (±2.77)	43.27 (±2.52)	29.02 (±3.24)
- Gamma	15.88 (±3.62)	48.45 (±2.79)	45.66 (±2.52)
- Omicron	5.92 (±3.92)	10.50 (±2.10)	7.45 (±1.64)
Absence of neutralizing antibodies against the variants, N (%)			
- Wild-type	42 (46.15%)	1 (0.98%)	6 (6%)
- Alpha	20 (21.74%)	6 (5.88%)	2 (2%)
- Delta	45 (49.45%)	4 (3.92%)	12 (12%)
- Gamma	40 (43.96%)	7 (6.86%)	6 (6%)
- Omicron	70 (76.92%)	43 (42.16%)	56 (56%)

## Data Availability

Data is unavailable due to privacy or ethical restrictions.
